# DOAC drug interactions management resource

**DOI:** 10.1177/17151635221116100

**Published:** 2022-09-20

**Authors:** Ayush Chadha, Micheal Guirguis, Tammy J. Bungard

**Affiliations:** Faculty of Pharmacy and Pharmaceutical Sciences; Faculty of Pharmacy and Pharmaceutical Sciences; Department of Medicine, University of Alberta and Pharmacy Services, Drug Utilization and Stewardship, Alberta Health Services, Edmonton, Alberta; the Division of Cardiology

## Background

**Figure 1:**
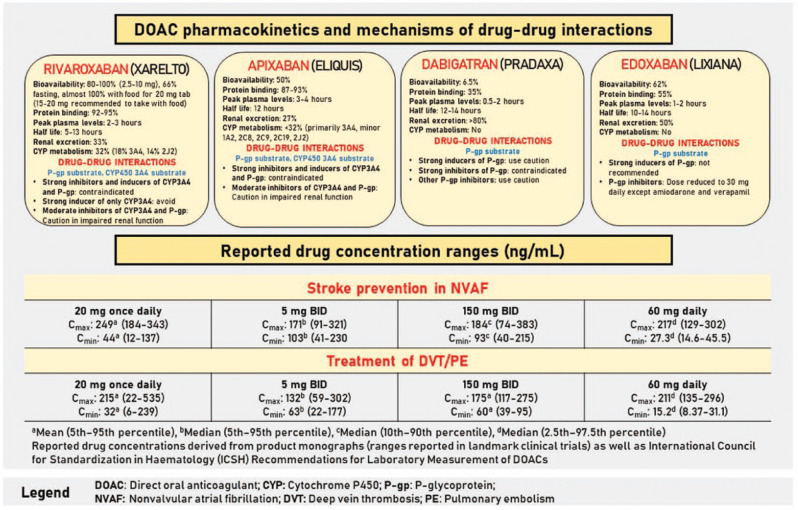
Overview of direct oral anticoagulants^1–6^

Over the past decade, direct oral anticoagulants (DOACs; apixaban, dabigatran, edoxaban and rivaroxaban) have offered many advantages over traditional therapy with warfarin ± low-molecular-weight heparins (LMWHs). The DOACs have established dosing without the need for coagulation monitoring as well as a quick onset (C_max_ at 1-4 hours) and offset (half-lives ranging from 9-14 hours for patients with normal renal function), thereby eliminating the need for bridging with LMWHs (Figure 1).^1-5^ Moreover, DOACs have fewer drug-drug interactions (DDIs) relative to warfarin; however, as the use of DOACs continues to increase in clinical practice, more information surrounding DOAC DDIs is necessary to make timely clinical decisions.

Pathways relevant to DOAC DDIs encompass the cytochrome P450 system (focusing on 3A4), as well as the P-glycoprotein (P-gp) transport system.^7^ Rivaroxaban and apixaban are substrates for P-gp and (in part) metabolized by CYP 3A4. Subsequently, rivaroxaban and apixaban DDIs must strongly affect both P-gp and CYP 3A4; the clinician should ensure a patient is not on 2 concomitant drugs that affect CYP 3A4 and P-gp separately, as these combined DDIs could cause significant changes in DOAC concentrations. In contrast, dabigatran and edoxaban are affected only by strong inhibitors/inducers of P-gp, as they lack metabolism by the CYP enzyme. The P-gp impact is within the gastrointestinal tract; hence, to minimize the P-gp DDI, dabigatran or edoxaban may be administered 2 hours prior to the interacting agent.^4^ Notably, all DOACs have a component of renal elimination (dabigatran > edoxaban > rivaroxaban > apixaban), and while progressive renal dysfunction will result in elevated DOAC concentrations, this elimination is not a direct mechanism of DDIs.^2-5^

At this time, there is limited clinical pharmacokinetic (PK)/pharmacodynamic (PD) data to quantify the clinical impact of specific DOAC DDIs. DDIs of this nature (i.e., P-gp or CYP 450) are highly variable because of the timing of the induction/inhibition turnover as well as the strength (mild, moderate or strong) of the interaction.^8^ In addition, there is inherent intersubject variability of 30% for concentrations of dabigatran, edoxaban and apixaban, with rivaroxaban reaching 40% for PK parameters.^9^ In addition, reported ranges of DOAC concentrations assessed in subgroups of clinical trials demonstrate variability in peak/trough ratios of nearly 1.6-fold.^2-5^ With this in mind, DDIs that alter DOAC concentrations of 30% to 40% often still result in DOAC concentrations falling within these reported concentration ranges. Subsequently, when regulators consider providing advice surrounding DDIs, within the context of high PK/PD variability, general recommendations are often to avoid these combinations; specifically, regulators contraindicate DOACs for DDIs with inducing agents (for fear of thrombotic events) and recommend use with caution and assess other factors that may warrant avoidance when an inhibitor is the interacting culprit.

Limited, if any, data provide a comparison of DDIs between the DOACs. Unique to edoxaban are recommendations for dose reduction (from 60 to 30 mg daily) in the presence of P-gp inhibitors (except amiodarone and verapamil), with certain drugs listed based on clinical trial protocols or product monograph content.^5,10^ As the front-line clinician continues to manage more complex clinical scenarios with consideration of DOAC use, a summary of available literature specific to DOAC DDIs is necessary, given there may be no or conflicting information for drug interactions. As such, our purpose is to provide a tool that differentiates DDIs across the 4 DOACs specific to agents commonly prescribed for patients with cardiovascular disease, with a description of available data to support the same.

## Development of the practice tool

To create the practice tool, a systematic approach was used to collate data from both product monographs and peer-reviewed literature available for DDIs with the DOACs. As conflicting information was identified across multiple sources, we streamlined our approach. First, a general table of drugs known to be CYP 3A4 and P-gp inhibitors and inducers was created using data from LexiComp and was cross-checked using the Food and Drug Administration (FDA) database where inconsistencies arose.^11,12^ Following this, all possible medication interactions were entered into the Lexi-Interact database—the one most commonly used by our clinical pharmacists.^13^ As most information was general in nature and based on a theoretical interaction, a formal search of the literature was then completed using the OVID database searching both MEDLINE (back to 1946) and Embase (back to 1974) on May 14, 2021, using the following search strategy: search term 1: “Dabigatran or Pradaxa or Apixaban or Eliquis or Rivaroxaban or Xarelto or Edoxaban or Lixiana or DOAC* or direct oral acting anticoagulant* or NOAC* or novel oral acting anticoagulant*” and search term 2: “Drug interaction* or Drug-drug interaction* or medication interaction*”. A total of 182 articles were identified and included if they demonstrated area under the curve (AUC) data or any clinical evidence (either drug concentrations or clinical outcomes) of a DDI. Among included articles, citations were also reviewed for relevant literature. Based on available data, recommendations for concomitant use with a DOAC (Table 1) were classified as follows:

- Green: No interaction or clinically nonsignificant interaction—no effect on pharmacokinetics
- Green/yellow: Use together with caution; limited data suggest either increased major bleeding or altered drug concentrations
- Yellow: Use with caution as either:
  - a theoretical/documented interaction that would affect DOAC concentration,
  - product monograph recommendation to use with caution, or
  - for edoxaban, recommendation to reduce dose (signified with ↓ dose)
- Yellow/red: Concomitant use is not recommended; limited data may support use
- Red: Avoid combination, may use only if DOAC concentrations are assessed as either:
  - theoretical/documented interaction that affects DOAC concentration or
  - product monograph recommendation to avoid or contraindicate, implies expected drug concentrations exceed the observed and acceptable variability

**Table 1:** DOAC drug interaction tool

Antiarrhythmic agents						
	Substrate	DDI mechanism	R	A	D	E
**Amiodarone**	3A4	Moderate 2C9 inhibitor Weak 3A4, 2D6 inhibitor P-gp inhibitor	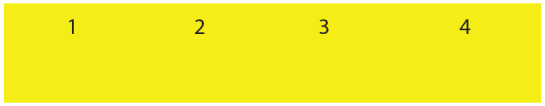			
**Dronedarone**	3A4	Moderate 3A4 inhibitor P-gp inhibitor	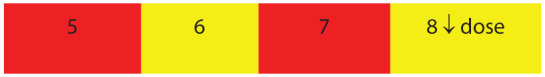			
**Propafenone**	3A4, 2D6	P-gp inhibitor	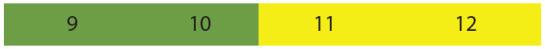			
**Quinidine**	3A4, P-gp	Weak 3A4 inhibitor P-gp inhibitor	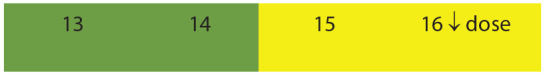			
Antibacterial agents						
	Substrate	DDI mechanism	R	A	D	E
**Azithromycin**	3A4	P-gp inhibitor	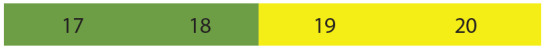			
**Ciprofloxacin**	P-gp	Strong 1A2 inhibitor Moderate 3A4 inhibitor	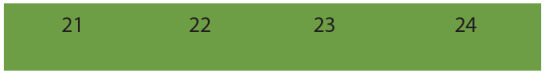			
**Clarithromycin**	3A4	Strong 3A4 inhibitor P-gp inhibitor	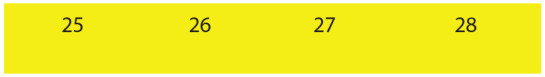			
**Erythromycin**	3A4, P-gp	Moderate 3A4 inhibitor P-gp inhibitor	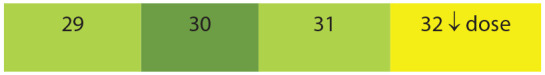			
**Rifampicin**	P-gp	Strong 3A4, 2C19 inducer Weak 2C9, 1A2 inducer P-gp inducer	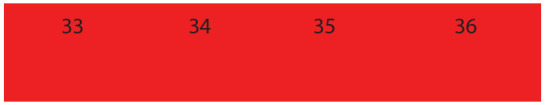			
Antidepressants						
	Substrate	DDI mechanism	R	A	D	E
**SSRI**	Pharmacodynamic		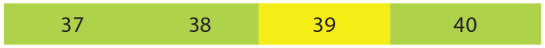			
**SNRI**		Pharmacodynamic	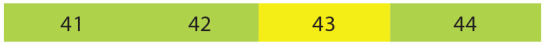			
Antiepileptic agents						
	Substrate	DDI mechanism	R	A	D	E
**Carbamazepine**	3A4, 2C8	Strong 3A4 inducer Weak 2C9/1A2 inducer P-gp inducer	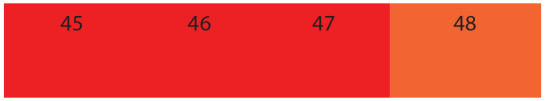			
**Phenobarbital**	2C19, 2C9	Strong 3A4 inducer Weak 2C9/1A2 inducer 2C19/2C9 substrate	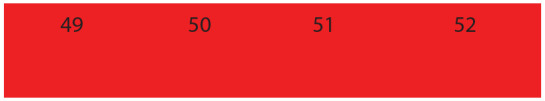			
**Phenytoin**	2C19, 2C9, 3A4	Strong 3A4 inducer Weak 1A2 inducer P-gp inducer	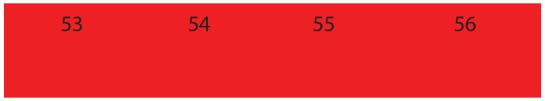			
**Other** (lamotrigine, levetiracetam, valproic acid)			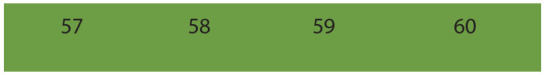			
Antiplatelet agents						
	Substrate	DDI mechanism	R	A	D	E
**Aspirin**	2C9	Pharmacodynamic	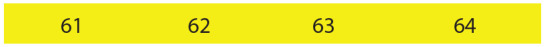			
**Clopidogrel**	2C19, 3A4	Moderate 2C8 inhibitor Pharmacodynamic	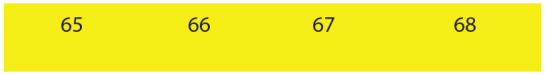			
**Ticagrelor**	3A4	P-gp inhibitor Pharmacodynamic	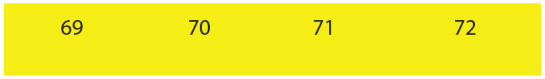			
Azole antifungal agents						
	Substrate	DDI mechanism	R	A	D	E
**Fluconazole**		Strong 2C19 inhibitor Moderate 3A4/2C9 inhibitor	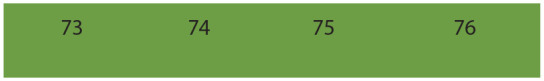			
**Itraconazole**	3A4	Strong 3A4 inhibitor P-gp inhibitor	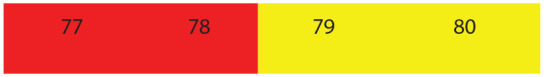			
**Ketoconazole**	3A4	Strong 3A4 inhibitor Weak 2C19/2C8 inhibitor P-gp inhibitor	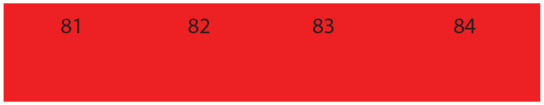			
**Posaconazole**	3A4	Strong 3A4 inhibitor	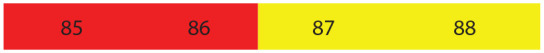			
**Voriconazole**	2C19	Strong 3A4 inhibitor Weak 2B6, 2C9, 2C19 inhibitor	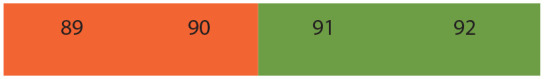			
Beta-blockers						
	Substrate	DDI mechanism	R	A	D	E
**Carvedilol**		P-gp inhibitor	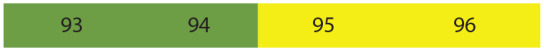			
**Other** (atenolol, bisoprolol, labetalol, metoprolol, nadolol, propranolol, sotalol, timolol)			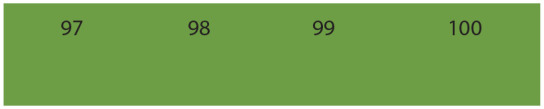			
Cardiotonic glycosides						
	Substrate	DDI mechanism	R	A	D	E
**Digoxin**	3A4, P-gp		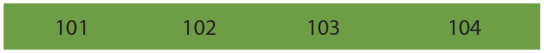			
Immunosuppresants						
	Substrate	Inhibitor	R	A	D	E
**Cyclosporine**	3A4, P-gp	Weak 3A4/2C9 inhibitor P-gp inhibitor	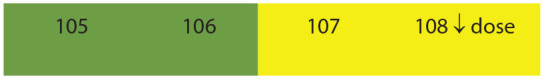			
**Tacrolimus**	3A4, P-gp	P-gp inhibitor	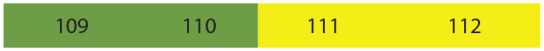			
Lipid-lowering agents						
	Substrate	DDI mechanism	R	A	D	E
**Lovastatin**	3A4, P-gp		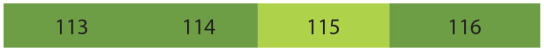			
**Simvastatin**	3A4, P-gp		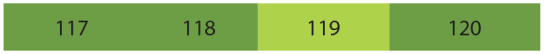			
**Other** (atorvastatin, rosuvastatin, fluvastatin, pravastatin)			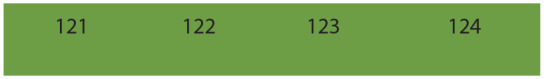			
Nonsteroidal anti-inflammatory drugs						
	Substrate	DDI mechanism	R	A	D	E
**Naproxen**	2C9, 1A2	Pharmacodynamic	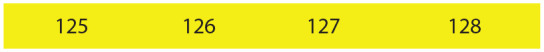			
**Other** (ibuprofen, diclofenac, ibuprofen, indomethacin, ketorolac, meloxicam)			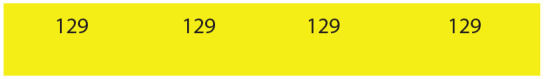			
Proton pump inhibitors						
	Substrate	DDI mechanism	R	A	D	E
**Esomeprazole**	3A4, 2C19	Weak 2C19 inhibitor Increase gastric pH	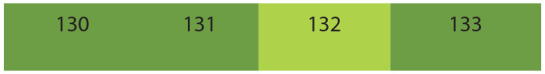			
**Omeprazole**	3A4, 2C19	Weak 2C19 inhibitor Increase gastric pH	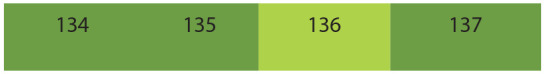			
**Pantoprazole**	3A4, 2C19	Increase gastric pH	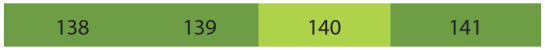			
**Other** (dexlansoprazole, lansoprazole, rabeprazole)		Increase gastric pH	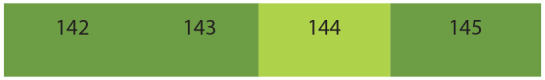			
SELECTIVE CALCIUM CHANNEL BLOCKERS						
	Substrate	Inhibitor	R	A	D	E
**Diltiazem**	3A4, 2C9, P-gp	Moderate 3A4 inhibitor	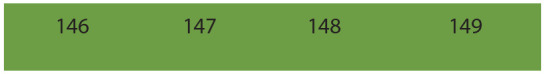			
**Verapamil**	3A4, 1A2, 2C9, P-gp	Moderate 3A4 inhibitor Weak 1A2 inhibitor P-gp inhibitor	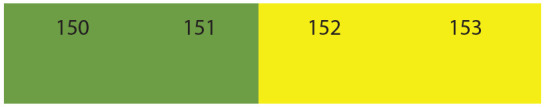			
**Other** (felodipine, nifedipine, amlodipine)			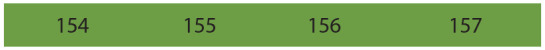			

Numbers in this table refer to interaction details described below. Disclaimer: To the best of our knowledge, the data in the table are an accurate summary of the published data up to July 2021. See full disclaimer at the end of the article.
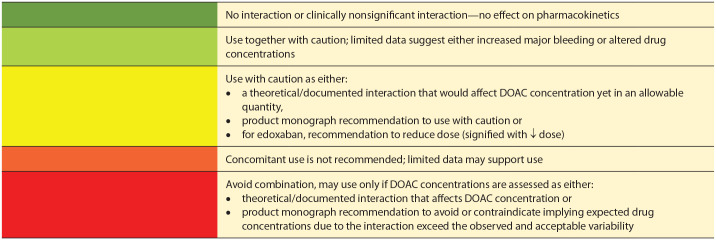

DOAC, direct oral anticoagulant; DDI, drug-drug interaction; R, rivaroxaban; A, apixaban; D, dabigatran; E, edoxaban; P-gp, P-glycoprotein; MB, major bleeding; GIB, gastrointestinal bleeding; PM, product monograph; ICH, intracerebral haemorrhage; SSRI, selective serotonin reuptake inhibitor; SNRI, serotonin–norepinephrine reuptake inhibitor.

**Interaction details:**

1. Rivaroxaban: no ↑ in MB (ROCKET-AF clinical trial^2,14^); ↑ MB (3 retrospective cohorts^15–17^); predicted ↑ in rivaroxaban area under the curve (AUC) by 37% (in silico study^18^)

2. Apixaban: ↓ MB compared with warfarin independent of amiodarone use (subanalysis of ARISTOLE^19^); ↑ MB (2 retrospective cohorts^15,17^); apixaban 5 mg bid + amiodarone 200 mg daily with hemopericardium (1 case report^20^); probable ↑ AUC by 30% and C_max_ by 40%^3^

3. Dabigatran: ↑ MB (1 retrospective cohort^15^), dabigatran 75 mg bid + amiodarone 200 mg daily with rectal bleeding –↓ renal function with dabigatran trough concentration at 5600 ng/mL (1 case report^21^); single dose of amiodarone 600 mg ↑ AUC by 60% and C_max_ by 50%^4^

4. Edoxaban: single dose of edoxaban 60 mg and amiodarone 400 mg daily × 4 days with ↑ in AUC by 40% and C_max_ by 66% (clinical trial in 30 healthy volunteers^5,22^)

5. Rivaroxaban: ↑ overall bleeding and GIB (2 retrospective cohorts^17,23^); + no ↑ MB (1 retrospective cohort^15^); PM not recommended^2^

6. Apixaban: no ↑ overall bleeding (1 retrospective cohort^23^); no ↑ MB (2 retrospective cohorts^15,24^); ↑ overall bleeding (1 retrospective cohort^17^); probable ↑ in AUC by 30% and C_max_ by 40% (based on diltiazem^3^)

7. Dabigatran: ↑ GIB (1 retrospective cohort^23^); no ↑ MB (1 retrospective cohort^15^); single and multiple doses of dronedarone 400 mg ↑ AUC by 114%-136% and C_max_ by 87%-125%^4^

8. Edoxaban: single dose of edoxaban 60 mg and dronedarone 400 mg twice daily × 7 days with ↑ in AUC by 46% and C_max_ by 66% (clinical trial in 34 healthy volunteers^5,22^)

9. Rivaroxaban: no anticipated drug interaction

10. Apixaban: no anticipated drug interaction

11. Dabigatran: no clinical data—theoretical interaction^4^

12. Edoxaban: no clinical data—theoretical interaction^5^

13. Rivaroxaban: no anticipated drug interaction

14. Apixaban: no anticipated drug interaction

15. Dabigatran: dabigatran 150 mg bid + dextromethorphan 20 mg/quinidine 10 mg bid resulting in lower GIB in a patient with acute kidney injury and ↑ thrombin time despite several doses of idarucizumab (1 case report^25^); ↑ in AUC by 53%—product monograph recommends separating administration of dabigatran by at least 2 hours before quinidine^4^

16. Edoxaban: single dose of edoxaban 60 mg and quinidine 300 mg × 2 days, ↑ in AUC by 77% and C_max_ by 85% (clinical trial in 42 healthy volunteers^22^)

17. Rivaroxaban: no anticipated drug interaction

18. Apixaban: no anticipated drug interaction

19. Dabigatran: no clinical data—theoretical interaction^4^

20. Edoxaban: no clinical data—theoretical interaction^5^

21. Rivaroxaban: no anticipated drug interaction

22. Apixaban: no anticipated drug interaction

23. Dabigatran: no anticipated drug interaction

24. Edoxaban: no anticipated drug interaction

25. Rivaroxaban: ↑ MB compared with either azithromycin or no clarithromycin use (1 elderly cohort^26^); no difference when used for *Helicobacter pylori* treatment combined erythromycin and clarithromycin (1 retrospective cohort^15^); rivaroxaban 20 mg daily + clarithromycin 500 mg twice daily resulting in ICH and rivaroxaban trough concentration of 537 ng/mL (1 case report^27^); single dose of rivaroxaban 10 mg daily and clarithromycin 500 mg twice daily ↑ AUC by 50% and C_max_ by 40% (clinical trial in 16 healthy volunteers^2,28^)

26. Apixaban: ↑ MB compared with either azithromycin or no clarithromycin use (1 elderly cohort^26^); ↓ MB when used for *H. pylori* treatment combined erythromycin and clarithromycin (1 retrospective cohort^15^) ↑ in AUC by 60% and C_max_ by 30%^3^

27. Dabigatran: ↑ MB compared with either azithromycin or no clarithromycin use (1 elderly cohort^26^); ↓ MB when used for *H. pylori* treatment combined erythromycin and clarithromycin (1 retrospective cohort^15^); single dose of dabigatran 300 mg and 500 mg clarithromycin twice daily ↑ AUC by 49% and C_max_ by 60% (clinical trial in 10 healthy volunteers^29^); coadministration of 500 mg bid clarithromycin with dabigatran ↑ in AUC by 19% and C_max_ by 15%^4^

28. Edoxaban: no clinical data – theoretical interaction^5^

29. Rivaroxaban: ↓ MB when used for *H. pylori* treatment combined erythromycin and clarithromycin (1 retrospective cohort^15^); erythromycin 500 mg tid and rivaroxaban ↑ in AUC by 30%^2^

30. Apixaban: ↓ MB when used for *H. pylori* treatment combined erythromycin and clarithromycin (1 retrospective cohort^15^)

31. Dabigatran: ↓ MB when used for *H. pylori* treatment combined erythromycin and clarithromycin (1 retrospective cohort^15^)

32. Edoxaban: single dose of edoxaban and erythromycin 500 mg qid for 8 days ↑ AUC by 85% and C_max_ by 68%^5^

33. Rivaroxaban: rivaroxaban 20 mg daily and rifampicin 150 mg bid leading to a fatal pulmonary embolism (PE) with peak rivaroxaban concentration at 178 ng/mL (1 case report^30^; rivaroxaban + rifampicin [doses not specified] ↓ AUC by 50%^2^)

34. Apixaban: coadministration of rivaroxaban + rifampicin 600 mg daily ↓ AUC by 54% and C_max_ by 42%^3^

35. Dabigatran: rifampicin 600 mg × 7 days + dabigatran ↓ AUC by 66% and C_max_ by 67%^4^

36. Edoxaban: single dose of edoxaban 60 mg and rifampicin 600 mg × 7 days ↓ AUC by 34% with no change in C_max_ (clinical trial in 32 healthy volunteers^5,31^)

37. Rivaroxaban: numerically ↑ MB in rivaroxaban and warfarin groups with SSRI vs without (subanalysis of ROCKET-AF^32^); ↑ MB with DOACs, but a secondary analysis with individual DOACs found no statistically significant interaction of rivaroxaban with SSRI (1 case-control study^33^)

38. Apixaban: apixaban coadministered with SSRI/SNRI did not show a significant ↑ MB compared with those on apixaban alone (cohort study^34^); ↑ MB risk with DOAC + SSRI vs no SSRI^3,35^

39. Dabigatran: ↑ MB with DOACs, a secondary analysis with individual DOACs found statistically significant interaction of rivaroxaban with SSRI (1 case-control study^34^); ↑ MB with dabigatran and warfarin with SSRI vs without (drug information manufacturer^4^)

40. Edoxaban: theoretical ↑ MB risk (not in other DOAC studies)

41. Rivaroxaban: no DDI studies done, yet potential ↑ risk of MB identified in case reports and epidemiological studies—theoretical impact^2^

42. Apixaban: apixaban coadministered with SSRI/SNRI did not show a significant ↑ MB compared with those on apixaban alone (cohort study^34^); ↑ MB risk with DOAC + SNRI vs no SNRI^3,35^

43. Dabigatran: no DDI studies done, yet potential ↑ risk of MB identified in case reports and epidemiological studies—theoretical impact (PM)

44. Edoxaban: theoretical ↑ MB risk (not in other DOAC studies)

45. Rivaroxaban: rivaroxaban 20 mg/day + carbamazepine 900 mg/day with reduced rivaroxaban concentration <20 ng/mL with recurrent venous thromboembolism (VTE; case report^36^); PE after total knee replacement taking rivaroxaban 10 mg/day + carbamazepine 600 mg bid without rivaroxaban concentration (case report^37^); avoid use^2^

46. Apixaban: Transient ischemic attack with apixaban 5 mg bid + carbamazepine 400 mg/day with peak apixaban concentration 94 ng/mL (case report^38^); apixaban 5 mg bid + carbamazepine 400 mg/day with peak apixaban concentration 110 ng/mL and trough 64 ng/mL—concentrations higher than while not taking carbamazepine (case report^39^); titration of carbamazepine with apixaban 5 mg bid + carbamazepine 800 mg/day had apixaban trough concentration 30 ng/mL + peak 114 ng/mL, apixaban 10 mg bid + carbamazepine 1000 mg/day with repeat trough/peak of 41/99 ng/mL (case report^40^); avoid use^3^

47. Dabigatran: dabigatran 150 mg bid + carbamazepine dose not specified yielded reduced dabigatran concentration of <30 ng/mL (2 case reports^41^); avoid use^4^

48. Edoxaban: edoxaban 60 mg/day + carbamazepine 400 mg/day with reference range edoxaban of peak 199 ng/mL after 2 weeks and 236 ng/mL after 4weeks (1 case report^38^); avoid use^5^

49. Rivaroxaban: theoretical interaction—no clinical data, avoid use^2^

50. Apixaban: cardioembolic stroke with apixaban 5 mg bid + “low-dose phenobarbital” with trough apixaban concentration of 89 ng/mL (1 case report^42^); avoid use^3^

51. Dabigatran: dabigatran + phenytoin or phenobarbital resulted in median corrected trough steady state >3 standard deviations below cohort mean (1 cohort study^42^); dabigatran 150 mg bid + “low-dose phenobarbital” had cardioembolic stroke after 3 months (no dabigatran concentration; 1 case report^43^)

52. Edoxaban: theoretical interaction—no clinical data; avoid use^5^

53. Rivaroxaban: rivaroxaban 15 mg bid + phenytoin 300 mg/day had peak rivaroxaban concentration of 70 ng/mL and 90 ng/mL (low), switched to dabigatran 150 mg bid with clinical improvement and thrombin time >180 seconds 4 hours postdose (1 case report^44^); avoid use^2^

54. Apixaban: theoretical interaction—no clinical data, avoid use^3^

55. Dabigatran: dabigatran + phenytoin or phenobarbital resulted in median corrected trough steady state >3 standard deviations below cohort mean (1 cohort study^42^); dabigatran 150 mg bid + phenytoin 300 mg/day with undetectable dabigatran concentration (case report^45^); left atrial thrombus with dabigatran 150 mg bid + phenytoin 300 mg/day, no dabigatran concentration noted (case report^46^)

56. Edoxaban: theoretical interaction—no clinical data (PM); avoid use^5^

57. Rivaroxaban: no anticipated drug interaction

58. Apixaban: no anticipated drug interaction

59. Dabigatran: no anticipated drug interaction

60. Edoxaban: no anticipated drug interaction

61. Rivaroxaban: ↑ MB; no clinically significant pharmacokinetic (PK) interaction with aspirin 500 mg^2^

62. Apixaban: ↑ MB; no clinically significant PK interaction with aspirin 325 mg^3^

63. Dabigatran: ↑ MB; no PK data available^4^

64. Edoxaban: ↑ MB; coadministration of aspirin 100 mg or 325 mg and edoxaban ↑ AUC by 32% and C_max_ by 35%^5^

65. Rivaroxaban: ↑ MB; clopidogrel 75 mg daily + single dose of rivaroxaban had no effect on PK^2^

66. Apixaban: ↑ MB, no changes in PK with clopidogrel 75 mg daily^3^

67. Dabigatran: ↑ MB, ↑ C_max_ by 30%-40% with loading dose of 300 or 600 mg clopidogrel^4^

68. Edoxaban: ↑ MB, no PK data^5^

69. Rivaroxaban: ↑ MB; no PK data; PM states not recommended^2^

70. Apixaban: ↑ MB; no PK data; PM states not recommended^5^

71. Dabigatran: ↑ MB; PK data reports an ↑ in AUC by 26%-49% and C_max_ by 24%-65%; PM states not recommended^4^

72. Edoxaban: No data, concurrent use not recommended by manufacturer due to bleeding risk^5^

73. Rivaroxaban: ↑ MB (retrospective cohort^15^); rivaroxaban 20 mg daily + fluconazole 400 mg/day × 6 days ↑ AUC by 40%^2,28^

74. Apixaban: ↑ MB (retrospective cohort^15^)

75. Dabigatran: ↑ MB (retrospective cohort^15^)

76. Edoxaban: no anticipated drug interaction

77. Rivaroxaban: no ↑ MB (combination of itraconazole, ketoconazole, posaconazole, voriconazole; retrospective cohort^15^); potential ↑ rivaroxaban concentration by 160%^2^

78. Apixaban: theoretical interaction—no data; avoid use per PM^3^

79. Dabigatran: no ↑ MB (combination of itraconazole, ketoconazole, posaconazole, voriconazole; retrospective cohort^15^); may ↑ dabigatran exposure, use with caution per PM^4^

80. Edoxaban: PM use with caution; in VTE trials, the dose was reduced to 30 mg daily^5^

81. Rivaroxaban: no ↑ MB (combination of itraconazole, ketoconazole, posaconazole, voriconazole; retrospective cohort^15^); ↑ AUC by 160% and C_max_ by 70%^2^

82. Apixaban: single dose of apixaban 10 mg and ketoconazole 400 mg/day ↑ AUC by 100% and C_max_ by 60% (clinical trial in 20 healthy volunteers^3,47^)

83. Dabigatran: no ↑ MB (combination of itraconazole, ketoconazole, posaconazole, voriconazole; retrospective cohort^15^); single and multiple oral doses of ketoconazole 400 mg daily ↑ AUC by 138%-153% and ↑ C_max_ by 135%-149%^4^

84. Edoxaban: single dose of edoxaban 60 mg and ketoconazole 400 mg/day ↑ AUC by 87% and C_max_ by 89%, decrease dose per PM (clinical trial in 37 healthy volunteers^5,48^)

85. Rivaroxaban: no ↑ MB (combination of itraconazole, ketoconazole, posaconazole, voriconazole; retrospective cohort^15^); may ↑ rivaroxaban concentration by 160%, which ↑ bleeding risk^2^

86. Apixaban: avoid use per PM—may ↑ exposure by twofold^3^

87. Dabigatran: no ↑ MB (combination of itraconazole, ketoconazole, posaconazole, voriconazole; retrospective cohort^15^); may ↑ exposure^4^

88. Edoxaban: no clinical data—may ↑ exposure^5^

89. Rivaroxaban: no ↑ MB (combination of itraconazole, ketoconazole, posaconazole, voriconazole; retrospective cohort^15^)—may ↑ exposure based on extrapolation with other azoles

90. Apixaban: contraindicated per PM—may ↑ exposure by twofold based on extrapolation with other azoles^3^

91. Dabigatran: no ↑ MB (combination of itraconazole, ketoconazole, posaconazole, voriconazole; retrospective cohort^15^

92. Edoxaban: no anticipated drug interaction

93. Rivaroxaban: no anticipated drug interaction

94. Apixaban: no anticipated drug interaction

95. Dabigatran: no data—theoretical interaction with P-gp inhibition, P-gp inhibitor per Food and Drug Administration (FDA)^4,12^

96. Edoxaban: no data—theoretical interaction with P-gp inhibition, P-gp inhibitor per FDA^5,12^

97. Rivaroxaban: no anticipated drug interaction

98. Apixaban: no anticipated drug interaction, ↓ in AUC by 15% and C_max_ by 18% of apixaban when coadministered with atenolol^3^

99. Dabigatran: no anticipated drug interaction

100. Edoxaban: no anticipated drug interaction

101. Rivaroxaban: no mutual PK interactions between digoxin and rivaroxaban^2^

102. Apixaban: no dose adjustment is required^3^

103. Dabigatran: no PK interaction observed—no dose adjustment required per PM^3^

104. Edoxaban: no clinical data—PK data ↑ C_max_ of edoxaban 17% and ↑ C_max_ 28% of digoxin per PM^5^

105. Rivaroxaban: rivaroxaban 20 mg + dose-individualized oral regimen of cyclosporine ↑ AUC by 47% and C_max_ by 104% (clinical trial in 12 healthy volunteers^49^); no ↑ MB (retrospective cohort^15^); mean for trough rivaroxaban concentration 131.7 ng/mL with cyclosporine compared with mean for trough rivaroxaban concentration 20.3 ng/mL with tacrolimus (cohort study in 9 patients after liver transplant, 5 received cyclosporine and 4 received tacrolimus^50^); all but 2 patients (both with renal dysfunction) had trough rivaroxaban concentration <137 ng/mL (upper limit of reported range; prospective observational study in 11 patients with orthostatic heart transplant, 8 received cyclosporine and 3 received tacrolimus^51^); no ↑ MB (dabigatran *n* = 9, rivaroxaban *n* = 17, apixaban *n* = 1, cyclosporine *n* = 2, tacrolimus *n* = 25; retrospective observational study^52^)

106. Apixaban: single dose of apixaban 10 mg and cyclosporine 100 mg daily × 3 days ↑ AUC by 20% and C_max_ by 43% (clinical trial in 12 healthy volunteers^53^); ↑ in MB (retrospective cohort^15^)

107. Dabigatran: ↑ in MB (retrospective cohort^15^); no ↑ MB among combined DOACs (dabigatran *n* = 9, rivaroxaban *n* = 17, apixaban *n* = 1, cyclosporine *n* = 2, tacrolimus *n* = 25) yet both MBs were taking dabigatran (retrospective observational study^52^); may be expected to ↑ systemic exposure to dabigatran and should be used with caution (theoretical^4^)

108. Edoxaban: cyclosporine 500 mg with a single dose of edoxaban 60 mg ↑ edoxaban AUC by 73% and C_max_ by 74% (clinical trial in 33 healthy volunteers^48^)

109. Rivaroxaban: No bleeding or thrombotic events, trough rivaroxaban concentration of 30-63 ng/L and peak rivaroxaban concentration of 134-449 ng/mL with limited variability in the 25^th^ to 75^th^ percentile range (prospective observational study in 8 renal transplant patients with stable renal function treated with tacrolimus ± everolimus^54^); mean for trough rivaroxaban concentration 131.7 ng/mL with cyclosporine compared with mean for trough rivaroxaban concentration 20.3 ng/mL with tacrolimus (cohort study in 9 patients after liver transplant, 5 received cyclosporine and 4 received tacrolimus^50^); all but 2 patients (both with renal dysfunction) had trough rivaroxaban concentration <137 ng/mL (upper limit of reported range; prospective observational study in 11 patients with orthostatic heart transplant, 8 received cyclosporine and 3 received tacrolimus^51^); no ↑ MB (dabigatran *n* = 9, rivaroxaban *n* = 17, apixaban *n* = 1, cyclosporine *n* = 2, tacrolimus *n* = 25; retrospective observational study^52^)

110. Apixaban: single dose of apixaban 10 mg and tacrolimus 5 mg daily × 3 days ↓ AUC by 22% and C_max_ by 13% (clinical trial in 12 healthy volunteers^53^)

111. Dabigatran: no ↑ MB among combined DOACs (dabigatran *n* = 9, rivaroxaban *n* = 17, apixaban *n* = 1, cyclosporine *n* = 2, tacrolimus *n* = 25) yet both MBs were taking dabigatran (retrospective observational study^52^); may be expected to ↑ systemic exposure to dabigatran and should be used with caution (theoretical^4^)

112. Edoxaban: no data—theoretical, P-gp inhibitor per FDA^5,12^

113. Rivaroxaban: no anticipated drug interaction

114. Apixaban: no anticipated drug interaction

115. Dabigatran: ↑ MB compared with other statins (case-control study^55^)

116. Edoxaban: no anticipated drug interaction

117. Rivaroxaban: no anticipated drug interaction

118. Apixaban: no anticipated drug interaction

119. Dabigatran: ↑ MB compared with other statins (case-control study^55^)

120. Edoxaban: no anticipated drug interaction

121. Rivaroxaban: no anticipated drug interaction, PM notes no drug interaction with atorvastatin^2^

122. Apixaban: no anticipated drug interaction

123. Dabigatran: no anticipated drug interaction, ↓ in AUC by 20% of dabigatran when coadministered with atorvastatin^4^

124. Edoxaban: no anticipated drug interaction, ↓ in AUC and C_max_ by 15% of edoxaban when coadministered with atorvastatin^5^

125. Rivaroxaban: ↑ MB; coadmininstration of naproxen and rivaroxaban did not affect rivaroxaban PK; no clinically relevant prolongation of bleeding time observed when 500 mg naproxen was preadministered 24 hours before concomitant administration of single doses of rivaroxaban 15 mg^2^

126. Apixaban: ↑ MB; single dose of 500 mg naproxen led to ↑ in AUC by 50% and 60% ↑ in C_max_ of apixaban (recommends no dose adjustment but use caution^3^)

127. Dabigatran: ↑ MB^4^

128. Edoxaban: ↑ MB; coadmininstration of naproxen and apixaban did not affect edoxaban PK, ↑ bleeding time relative to either alone^5^

129. Diclofenac, ibuprofen, indomethacin, ketorolac, meloxicam—no PK data, pharmacodynamic interaction suspected^12^

130. Rivaroxaban: no anticipated drug interaction

131. Apixaban: no anticipated drug interaction

132. Dabigatran: concurrent proton pump inhibitor (PPI) administration ↓ trough dabigatran concentration and peak dabigatran concentration by 33% than without coadministration (clinical trial in 35 patients with nonvalvular atrial fibrillation [NVAF] 14 lansoprazole, 14 rabeprazole, 6 esomeprazole^56^); coadministration of PPIs with dabigatran ↓ AUC by 12.5% (PK analysis of RE-LY trial^57^)

133. Edoxaban: single dose of edoxaban and esomeprazole 40 mg once daily × 5 days had no effect on the AUC of edoxaban but the Cmax ↓ by 33%—no dose modification is necessary^5^

134. Rivaroxaban: single dose of rivaroxaban and multiple doses of omeprazole, geometric means for AUC and C_max_ were within 80%-125% range (clinical trial in 22 healthy volunteers^58^); coadministration of rivaroxaban and omeprazole did not affect rivaroxaban PK [(2)]

135. Apixaban: no anticipated drug interaction

136. Dabigatran: concurrent PPI administration ↓ trough dabigatran concentration and peak dabigatran concentration by 50% than without coadministration (prospective observational study in 31 hospitalized patients 9 omeprazole 10 pantoprazole 12 no PPI^59^); coadministration of PPIs with dabigatran ↓ AUC by 12.5% (PK analysis of RE-LY trial^57^)

137. Edoxaban: no anticipated drug interaction

138. Rivaroxaban: no anticipated drug interaction

139. Apixaban: no anticipated drug interaction

140. Dabigatran: concurrent PPI administration ↓ trough dabigatran concentration and peak dabigatran concentration by 50% than without coadministration (prospective observational study in 31 hospitalized patients 9 omeprazole 10 pantoprazole 12 no PPI^59^); single dose of dabigatran + pantoprazole ↓ AUC by 32% and C_max_ by 40% (clinical trial in 18 healthy volunteers^60^), dabigatran 150 mg bid + pantoprazole 40 mg bid ↓ the AUC and C_max_ by 20% compared with subjects not on pantoprazole (clinical trial in 36 healthy elderly volunteers^61^), coadministration of dabigatran + pantoprazole ↓ in AUC by 30%^4^

141. Edoxaban: no anticipated drug interaction

142. Rivaroxaban: no anticipated drug interaction

143. Apixaban: no anticipated drug interaction

144. Dabigatran: concurrent PPI administration ↓ trough dabigatran concentration and peak dabigatran concentration by 33% than without coadministration (clinical trial in 35 patients with NVAF 14 lansoprazole, 14 rabeprazole, 6 esomeprazole^56^); coadministration of PPIs with dabigatran ↓ bioavailability AUC by 12.5% (PK analysis of RE-LY trial^57^)

145. Edoxaban: no anticipated drug interaction

146. Rivaroxaban: rivaroxaban + diltiazem was not associated with ↑ bleeding (retrospective cohort^62^); no ↑ MB (retrospective cohort^15^); no ↑ in overall bleeding in patients treated with verapamil or diltiazem vs amlodipine or metoprolol with rivaroxaban (retrospective cohort^63^); ↑ in MB and ICH across both rivaroxaban and warfarin (analysis of data from clinical trial ROCKET AF^14^)

147. Apixaban: no ↑ in MB (retrospective cohort^15^); no ↑ in overall bleeding in patients treated with verapamil or diltiazem vs amlodipine or metoprolol with apixaban (retrospective cohort^63^); diltiazem 360 mg daily + apixaban led to ↑ in AUC by 40% and Cmax by 30%; no dose adjustment required, use with caution^3^

148. Dabigatran: ↑ in overall bleeding in patients treated with verapamil or diltiazem vs amlodipine or metoprolol (retrospective cohort^63^); no ↑ in MB (retrospective cohort^15^)

149. Edoxaban: no anticipated drug interaction

150. Rivaroxaban: concurrent verapamil + rivaroxaban ↑ AUC by 40% (clinical trial in 27 volunteers with normal or mildly impaired renal function^64^); no ↑ in overall bleeding in patients treated with verapamil or diltiazem vs amlodipine or metoprolol with rivaroxaban (retrospective cohort^63^); no ↑ in MB (retrospective cohort^15^); ↑ in MB and ICH across both rivaroxaban and warfarin (analysis of data from clinical trial ROCKET AF^14^)

151. Apixaban: no ↑ in overall bleeding in patients treated with verapamil or diltiazem vs amlodipine or metoprolol with apixaban (retrospective cohort^63^); no ↑ MB (retrospective cohort^15^)

152. Dabigatran: ↑ in overall bleeding in patients treated with verapamil or diltiazem vs amlodipine or metoprolol with dabigatran (retrospective cohort^63^); no ↑ MB (retrospective cohort^15^); coadminitration of 150 mg dabigatran once daily with verapamil (120 mg bid or 240 mg) resulted in variable ↑ of dabigatran AUC by 20%-150% and Cmax by 10%-180% depending on the timing (1 hour prior, concurrently, 2 hours after, steady state) of administration and the formulation (immediate or extended release) of verapamil used. Simultaneous initiation of treatment with dabigatran and verapamil should be avoided at all times. In all cases, to minimize potential interaction, dabigatran should be given at least 2 hours before verapamil. Use caution.^4^

153. Edoxaban: single dose of edoxaban 60 mg + extended release verapamil 240 mg daily for 11 days ↑ the AUC and Cmax by 53% (clinical trial in 34 healthy volunteers^5,22^)

154. Rivaroxaban: no anticipated drug interaction

155. Apixaban: no anticipated drug interaction

156. Dabigatran: no anticipated drug interaction

157. Edoxaban: no anticipated drug interaction

Inclusion of all actual or potential DDIs with DOACs was beyond the scope of our tool. As this tool was created for use by practitioners within an anticoagulation clinic having a thrombosis/cardiology-based practice, herbal supplements and drug classes such as (but not limited to) hormonal agents, monoclonal antibodies, tyrosine kinase inhibitors, intercalating agents and antimitotic agents were excluded, given they are not commonly encountered in our practice. As DDIs most relevant to the DOACs involve either P-gp or CYP 3A4, we also identified if potentially interacting medications were substrates of these pathways and to what extent (mild, moderate, severe). In doing so, we allow the clinician to extrapolate the potential impact that an inducer/inhibitor may have on these drug concentrations.

## Clinical management of DOAC DDIs

To effectively manage a potential/actual DDI with a DOAC, the clinician should consider individual patient characteristics and how these may have an impact on anticipated DOAC concentrations. For patients prescribed anticoagulants, the clinician should assess the risk of clotting vs bleeding to provide a basis for comfort in having the patient’s anticipated DOAC concentration on the higher vs lower end. Risk for clotting is specific to the indication for anticoagulant use; for some indications, validated risk scores are available (e.g., CHADS_2_ score for nonvalvular atrial fibrillation), whereas for others, such as venous thromboembolism, clinical factors such as the proximity/extensiveness of the clot are more helpful. Specific to bleeding risk, the clinician should contemplate factors that encompass patient history of bleeding, diseases of note (e.g., esophageal varices, diffuse diverticulitis) or drugs increasing risk (e.g., concomitant antiplatelet therapy). Knowledge of renal dysfunction and the impact on DOAC concentration should also be integrated into this assessment. Once done, the clinician should extrapolate a preference for having the DOAC concentration on the high end (assuming clot risk trumps bleeding risk) or the low end (assuming the opposite).

## Conclusion

This tool has been developed to assist clinicians in making decisions surrounding DOAC use. The clinician is encouraged to review the basis of the recommendation with available literature described, all drugs being administered andrenal function to gauge the overall impact on DOAC concentration. With this in mind, clinical judgement should dictate practice. ■
